# Results of a Primary Skin-Cancer-Prevention Campaign in Early Childhood on Sun-Related Knowledge and Attitudes in Southern Hungary

**DOI:** 10.3390/cancers13153873

**Published:** 2021-07-31

**Authors:** Zsuzsanna Horváth, Csernus A. Evelin, Péter Oláh, Rolland Gyulai, Zsuzsanna Lengyel

**Affiliations:** Department of Dermatology, Venerology and Oncodermatology, Medical School, University of Pécs, 7632 Pécs, Hungary; horvath.zsuzsanna2@pte.hu (Z.H.); csernus.evelin@pte.hu (C.A.E.); olah.peter@pte.hu (P.O.); gyulai.rolland@pte.hu (R.G.)

**Keywords:** primary prevention, children, daycare centers, kindergarten, ultraviolet radiation

## Abstract

**Simple Summary:**

Primary skin-cancer-prevention campaigns among young children are important as this is the age when individuals are developing behaviors. Our aim was to evaluate sun-protection knowledge and behavior among caregivers in daycare centers and kindergartens and to determine if educational lectures are positively influential. In daycare centers, we discovered that measures of sun protection (e.g., hat, sunscreen, and shaded areas) are more likely to be available when compared to kindergartens. Knowledge regarding sun safety has improved following our initial presentation, however, not significantly. Sun safety policies did not exist in any of the facilities, presenting an urgent need for their implementation.

**Abstract:**

Avoidance of ultraviolet (UV) exposure in early childhood is important for reducing the lifetime risk of developing skin cancer. The goal of the present prospective, multicenter pilot study was to assess the sun-protection practices in kindergartens and daycare centers and to evaluate sun protection knowledge and behavior among caregivers employed in the surveyed facilities. The study consisted of two parts. A baseline questionnaire was completed by the caregivers in relation to knowledge regarding basic sun protection and sun protection practices of the participating facilities. Afterward, a thirty-minute presentation was hosted in reference to this topic. Six months following the presentation, a follow-up questionnaire was distributed among the caregivers, evaluating the attitude-related and behavioral changes towards children. A total of 153 caregivers from five daycare centers (children between 6 months and 3 years of age) and sixteen kindergartens (children between 3 and 7 years of age) willfully participated in our study. According to our results, the main source of information regarding sun protection originated from different types of media. We found that staying in shaded areas and the use of protective clothing were not frequent in the facilities. Following our presentation regarding skin types and sunscreen use, protective measures improved, but not significantly (*p* = 0.222). The majority (92.31%) of caregivers distributed the information throughout their environment and also to parents. Sun protection knowledge is necessary; however, motivation among caregivers and parents and involvement of children is also relevant. Hence, a continuous, repetitive educational program regarding sun-smart behavior is deemed essential.

## 1. Introduction

The incidence of melanoma and non-melanoma cancers has shown a persistent increase worldwide in recent decades [1,2], placing an increasing burden on the health care system. Recently, incidence rates of melanoma were documented as high as 35 cases in 100,000 in Australia and around 14 cases in 100,000 in both the USA and UK [1]. In 2019, 24 new melanoma cases per 100,000 inhabitants were diagnosed in Hungary according to the National Health Insurance Fund of Hungary (NHIF) [3]. A recent study found significant changes in melanoma incidence trends in Hungary. Between 2011 and 2015, a significant mean annual increase was detected in the overall melanoma incidence, while between 2015 and 2019, a relevant mean annual decrease was seen. The authors assumed that joining the Euromelanoma campaign supported existing Hungarian seasonal screening programs to reach a wider population leading to the decline [3].

Solar and artificial UV exposure is the main risk factor for the development of melanoma and non-melanoma skin cancers. UV exposure in childhood and adolescence elevates the individual’s lifetime risk of developing skin cancer more than exposure in adulthood [4]. The majority of lifetime sun exposure occurs from early childhood to adolescent ages, and 50% to 80% of lifetime cumulative sun exposure occurs prior to 18 years of age [5]. In particular, early-childhood sun exposure and blistering sunburn prior to the age of 20 have been shown to be a determinant risk factor regarding malignant melanoma [6,7,8], and it may increase the risk by nearly two-fold [9]. Children spend more time outdoors, toddlers are harder to restrict to shaded areas during outdoor activities, and the skin of small children is more vulnerable to the effects of UV radiation (biological vulnerability) [10,11,12,13]. Other risk factors are also involved in skin tumor formation such as genetic predisposition, fair skin, the presence of atypical melanocytic naevi, and high numbers of melanocytic naevi (latter two in melanoma) [14,15,16]. Among all the risk factors, UV radiation is the only exogenous factor and therefore the one targeted for modification.

Since the 1980s, several health-education campaigns and skin-cancer-prevention programs have evolved worldwide [17]; however, few studies are available referencing the very young, pre-school-aged children [18,19]. As of this writing, no organized primary skin-cancer-prevention program exists in Hungary for pre-school-aged children.

Health education campaigns can be effective in terms of improving knowledge, attitude, and behavior among young children [20,21]. Adult health behavior is often established during childhood. According to recent publications, proper sun-protection habits of the mother led to less sun exposure among children [7,9,22,23].

The effectiveness of melanoma prevention is dependent upon how it is accepted by the population. Fun and at the same time deterrent examples are often used in campaigns [21,24,25,26]. Educational purposes can be achieved through environmental intervention (brochures, handouts, and books), presentations, and behavioral intervention (role play, and games), the latter of which has proven effective regarding children [15,26].

In many EU countries (including Hungary), the use of tanning booths is prohibited to minors [27]; however, there is a lingering misconception according to which acquiring a suntan indoors offers protection from skin cancer and the harmful effects of UV light [28]. We find it immensely important to educate children, parents, and caregivers, to set a positive example by shunning the use of artificial UV light.

All three stakeholders (caregivers, parents, and children) should be targeted to achieve effective implementation of sun-safe behavior [29]. A study in German childcare centers demonstrated a significant improvement in knowledge of sun-related issues among staff members and parents following a relatively brief training session. Children were also taught sun safety messages through a playful approach. The effectiveness regarding children’s learning was not assessed in the study, yet 82% of caregivers believed that children learned something beneficial regarding sun protection [30,31].

No data were available in reference to sun-protection policies and habits applied in Hungarian daycare centers and kindergartens when the study was designed. Therefore, our primary aim was to survey any existing sun-safety measures among the two types of institutions. Our secondary goal was to evaluate the knowledge of caregivers regarding sun-safety and whether educational presentations can improve such knowledge, since they spend eight to ten hours among our youngest ones five days out of a week.

## 2. Materials and Methods

### 2.1. Participating Facilities

The study was conducted in the year 2017 and included five daycare centers (children between 6 months and 3 years) and sixteen kindergartens (children between 3 and 7 years). All childcare institutions were located in the city of Pécs, in the southern part of Hungary, with a Mediterranean climate. The sunshine duration in Pécs between May and August varies between 245 and 289 h. The yearly average sunshine duration is 2080 h [32].

The kindergartens were all “green kindergartens”, implying that they follow an environmentally conscious education program. All facilities are financed by the city government and located in neighborhoods with average-income households.

Throughout our investigation, two self-administered questionnaires and a multi-media presentation were used, which were developed by the authors of this article. All institutions were personally visited by the authors. Participation of the staff members was voluntary and anonymous.

### 2.2. Questionnaires and Educational Intervention

The baseline questionnaire consisted of twenty-eight questions. The first questions (Q3-13) of this questionnaire evaluated the caregivers’ knowledge regarding basic sun protection information. The second part assessed the sun protection practices of the kindergarten or daycare center (e.g., the time frame, when children are taken outside during the summer, availability of shaded areas, and the use of sunscreen, sunhat, and sunglasses) and the knowledge of correct sunscreen use by caregivers (Figure S1).

Following our assessment of the first questionnaire, an approximately thirty-minute-long presentation was hosted by a dermatologist (one of the authors). The lecture highlighted information regarding the basics of the physical properties (ambient UV exposure via reflectance of UV radiation, glass, and water penetration), and the human biological effects of UV radiation including its cancerogenic and photoaging effects (highlighted with clinical pictures). The different skin types, UV index, knowledge regarding ozone depletion and sun protection skills were presented, along with a special emphasis in reference to shade seeking, wearing proper clothing (broad-brimmed hats, sunglasses, and long-sleeved clothing with ultraviolet protection factor—UPF) and the proper use of sunscreens. The importance of avoiding sunbeds, avoiding midday hours spent in direct sunlight, and successful primary prevention campaigns were also mentioned (SunSmart and SunWise programs).

Six months (including summer months) following our presentation, a follow-up questionnaire was distributed to the caregivers of the same institutions in early autumn, who participated in our presentations. This questionnaire evaluated the attitudinal and behavioral change in relation to children and of themselves concerning sun safety and the acquired knowledge regarding sun protection skills. Positive environmental changes in the proportion of shaded areas in surveyed institutions were also evaluated (Figure S2).

The tests were scored to determine knowledge regarding two topic areas prior to and following the presentation. Knowledge regarding skin types was measured by a match-test question in which correct answers were awarded with one point out of a maximum six points. Proper sunscreen application was scored with single choice test questions, and correct answers were also awarded one point. Questions regarding UV light properties were measured using single-choice test questions (Q3-6-7-8-9) in the first test and multiple-choice test in the second questionnaire (Q2). Correct answers were awarded with one point each, in which the percentage of correct answers was compared with various knowledge traits. The pre-test was filled out by 153 participants (57 daycare workers and 96 kindergarten employees). The post test was completed by staff members who attended the intervention, a total of 143 participants (56 daycare workers and 87 kindergarten employees). The total number of answerers depicted in the tables vary, since not all questions were answered by all participants.

### 2.3. Statistical Analyses

The responses were analyzed, and odds ratios were calculated. Samples were tested for normality using the Kolmogorov–Smirnov test, and hypotheses were tested with independent *t*-test, Mann–Whitney U test, and chi-square tests, as appropriate. A 95% confidence interval was used (*p* = 0.05) to determine statistical significance (*p*-values are also provided). 

## 3. Results

### 3.1. Baseline Questionnaire

One hundred and fifty-three female caregivers (daycare center *n* = 57, kindergarten *n* = 96) completed our first questionnaire. The mean age of the respondents of the first questionnaire was 46.5 years (21–62 years), (daycare centers, 43.5 years (21–60 years); kindergartens, 48.4 years (25–62 years)).

#### 3.1.1. Source of Information Regarding Sun Protection

Analysis of the results of the first questionnaire on basic sun protection knowledge revealed that virtually none of the participants received any form of education on sun protection during their training—not even in the “green kindergartens”.

**Figure 1:**
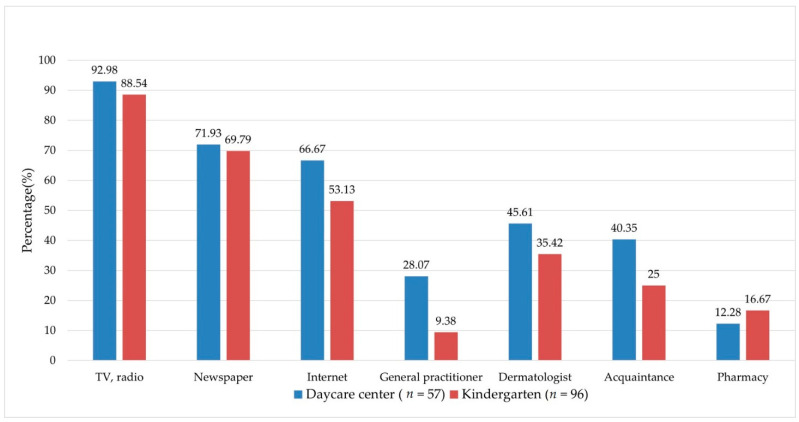
Information sources among caregivers concerning sun safety.

The most common (68.37%) source to gather information regarding sun protection was the different means of media (TV, radio, internet, and the newspaper). The caregivers employed in the nurseries were more likely to acquire information from a healthcare professional as compared to kindergartens (from general practitioners (GPs) 28.07% vs. 9.38%, from dermatologists 45.61% vs. 35.42%, respectively) (Figure 1).

#### 3.1.2. Knowledge Regarding UV Light Properties and Sun Protection

Correct answers were given by 75.8% of the caregivers to the questions on the effects of UV radiation on the skin, UV index, the time interval that should be avoided while exposed to direct sunlight, and sun protection practices. However, caregivers experienced difficulties determining the different skin types: only 32.0% (*n* = 49) of all caregivers were able to identify all of them. Caregivers employed in daycare centers scored better results: 42.11% reached maximum points, while in kindergartens it was 26%. In ascertaining the knowledge regarding sunscreen use, only 11.9% of all caregivers reached the maximum points allocated. A larger proportion of caregivers in the daycare centers (15.69%) achieved maximum points in this section of the questionnaire than caregivers in kindergartens (9.64%) (Table 1). The average knowledge regarding sun safety of caregivers in the daycare centers was slightly increased (mean of ranks: 72.3 in daycare centers vs. 64.5 in kindergartens), although the difference was not significant (*p* = 0.228) (not shown in table).

#### 3.1.3. Sun-Protection Practices in the Facilities

According to the directors of all institutions, no written sun protection policy existed in the facilities. Analysis of the sun-protection practices in the institutions revealed that children stay outdoors between 09:00 and 11:00 and between 15:00 and 17:00 h. Additionally, if the outdoor temperature rises above 30 °C, children stay inside. This practice is verbally communicated to the staff.

All centers named trees as the main source of shading within their own institutions. Almost all (98%) caregivers claimed that the institution yard is properly shaded but staff members only rated shade over play structures rather than the entire outdoor area. In contrast, the authors noted that most of these institutions lack properly shaded areas. On average, half of the entire outdoor area in the daycare centers was shaded, while in the kindergartens it was only one-third. However, daycare centers were smaller in size, and on average, the number of attending children is 50 or less, while 100–150 children are cared for in the surveyed kindergartens. In evaluating the availability of sun-protection measures in the facilities, we found that the prevalent measures used in sun protection among children were distributed as follows: in most of the centers only a few of the children wear sunglasses (daycare centers: 1.75% and kindergartens: 15.63%), a larger proportion of children have sunhats in the daycare centers (daycare centers: 89.74%, kindergartens 73.96%), and the availability of sunscreens are more common in the daycare centers (daycare centers: 68.42%, kindergartens 36.5%) (Table 2). The general availability of sunhat and sunscreen differed significantly between daycare centers and kindergartens (*p* < 0.001, chi-square test), and a similar difference is indicated in sunglasses availability as well (*p* = 0.007). Sunglasses are often considered hazardous to young children and their use is not encouraged; the latter was communicated by several of the staff members as a side note (Table 2). 

Nearly all caregivers help those in their care in putting on hats (100%) and aid in applying sunscreen (98.7%) when going outdoors, if indeed, and generally, the child comes equipped with his/her own personal hat and sunscreen. The majority of caregivers encourage parents to bring a hat and sunscreen to the child centers (sunhat 86.3% and sunscreen 88.9%).

**Figure 2:**
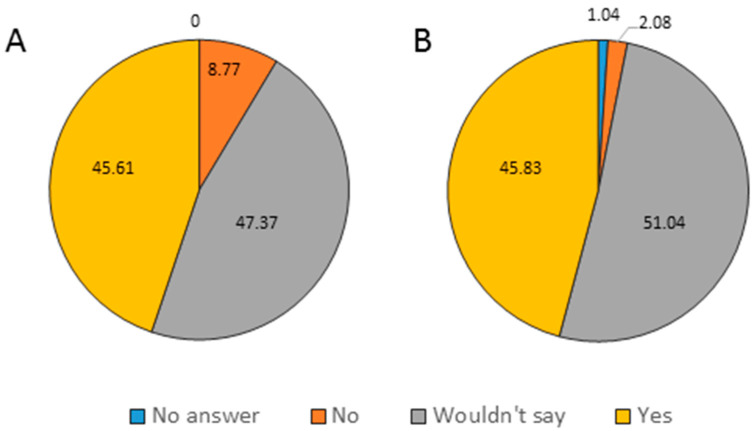
Availability of sunhat and sunscreen to fair-skinned children based on caregiver’s judgment in daycare centers (**A**) and kindergartens (**B**).

The authors were interested in knowing if children having skin types I and II are more likely to have access to sun-protection measures provided by their parents. Less than half of the staff members believed there is a correlation (daycare centers: 45.61%, kindergartens: 45.83%) (Figure 2).

Ninety-eight percent of all caregivers believe sun safety is important; however, the willingness to learn more regarding sun safety was only 65.4%.

### 3.2. Follow-Up Questionnaire

One hundred and forty-three caregivers (daycare centers *n* = 56, kindergartens *n* = 87) participated in our presentation and filled out the second questionnaire. The mean age of the responders of the second questionnaire was 43.7 years (22–60 years) (daycare centers, 39.8 years (22–57 years): kindergartens, 46.6 years (24–60 years)). Distinctly, 91.6% of all caregivers found the presentation educative and useful in the second questionnaire.

#### 3.2.1. Knowledge Regarding UV Light Properties, Sun Protection, and Changes in Behavior

A total of 80.4% of all caregivers achieved at least 60% correct answers in the second questionnaire regarding the properties and biological effects associated with UV light. The knowledge of the meaning of UV index was shown to be accurate, as 91.5% correctly responded (daycare center: 82.7% and kindergarten: 93.7%).

In general, caregivers scored higher levels of accuracy regarding questions about sunscreen use (14.7%), yet the improvement was not significant (baseline test 11.9%, *p* = 0.222). Additionally, an insignificant improvement was detected regarding the identification of the different skin types: 44.7% of all caregivers reached maximum points in the second questionnaire (baseline test 32%, *p* = 0.307) (Table 1).

A total of 40.6% (*n* = 58) of caregivers claimed to have changed their sun-protection habits, including giving up sunbathing, confining themselves to shady areas, using the appropriate amount of sunscreen, donning sunhats and sunglasses and/or protective clothing. A total of 43.1% (*n* = 25) of these caregivers changed one, two (27.6%, *n* = 16), three (22.4%, *n* = 13), and four (3.4%, *n* = 2) of their sun protection habits, respectively (Table 3).

Eighty-four (58.5%) caregivers did not alter their sun-protection habits, among those resolute in keeping to their former habits. Thirty (20.9%) reportedly had never tanned, and 27% (*n* = 39) continued sunbathing despite the newly acquired information. Fifteen (17.86%) caregivers did not specify the reason for their unaltered sun-protection habit; however, respondents claimed they kept doing it since they enjoy outdoors, including exposure to the sun, they have no history of sunburn, and make adequate use of sunscreens. Almost all caregivers stated that they shared the acquired information on sun safety with family, friends, and colleagues (92.3%). The majority of caregivers raised awareness in regard to parents highlighting the major factors of sun safety (68.5%).

#### 3.2.2. Sun-Protection Practices in the Facilities

The majority (95.8%) of caregivers in daycare centers and kindergartens both agreed that they consciously paid more attention to the sun safety of children, with an emphasis on restricting play to shaded areas of the yard and using sunhats and sunscreens.

All directors stated they were not able to develop the proportion of shaded area as large as they wanted. The most common cause was the lack of financial support.

## 4. Discussion

There are a substantial number of studies evaluating the effect of sun-protection programs targeting preschool-aged children and caregivers and showing favorable results in education and awareness [19,25,26,30,31], although there is a paucity of such programs according to literature data in Eastern Europe; therefore, the authors find it important to survey and help to develop conscious and sun-safe behavior in caregivers and small children.

This manuscript provides preliminary results of our primary skin cancer prevention program. Our primary prevention program sets the goal of educating the proper form of sun protection first among caregivers and later the parents of children aged between 6 months and to the end of the kindergarten years, as well as the goal of involving children in the learning process in a playful way. It is important that caregivers (including parents) in charge of the youngest children are thoroughly educated regarding sun protection since their actions and behaviors set the foundation for lifelong correct sun-protection practices for children within their care [11,17,23,33,34].

Additionally, recent studies underline the necessity of primary prevention awareness campaigns targeting children, since even adults in risk groups (sailors and agriculture employees) show a lack of information and disinterest in sun protection measures [35,36].

In our study, we found that no education program in reference to sun protection currently exists for training caregivers of preschool children in the surveyed facilities. However, similarly to previous studies [22,28], our results demonstrated that caregivers possessed relatively accurate knowledge regarding the biological effects of UV light. However, this premise does not necessarily translate into appropriate sun-safe behavior [37]. The knowledge level regarding different skin types improved following our presentation, but the difference was not significant. These results are in agreement with the German “Sun-Pass” project, on which the percentage of staff members naming the skin types correctly increased only slightly [31]. Daycare center caregivers achieved higher scores on the queries regarding sunscreen use and skin type. This insignificant difference may be explained by data that show that caregivers in daycare centers are more likely to acquire their information from doctors compared to employees in kindergartens. On the other hand, post-intervention scores regarding sunscreen use decreased in the daycare centers, which may be biased by the number of responders (51 pre-test vs. 56 post-test). Overall, the knowledge of sunscreen use did not significantly improve, suggesting the correct form of sunscreen use has yet to be taught, as has been previously emphasized in another study [38]. In our study, the average knowledge regarding sun safety of caregivers in the daycare centers showed a slight increase; however, the difference was not significant. Not being able to demonstrate a significant change after intervention, as shown by other studies [30,31], might have been influenced by the fact that the duration of time between the two tests was too long (6 months), and therefore recurring education is necessary within six months or less. As a part of an Italian primary prevention program among primary school children, parents were asked to complete pre- and post-intervention tests regarding the use of sun-protection measures. No significant difference was stated concerning the investigated factors, with the exception of a slight increase in the use of sunhats and sunglasses (elapsed time between the two tests was approx. 6 months). However, the authors were able to document a slight improvement regarding behavior and reduced sunburn rates over almost two decades [39].

Additionally, in our study, we found that less than half of the staff members believed that there is a correlation between sunscreen availability for fair-skinned children provided by their parents (daycare centers: 45.61%, kindergartens: 45.83%). Our results can be compared with a survey conducted in the USA involving parents of preschool-aged children; in that study, 72.6% of parents’ children who sunburn easily used sunscreen, compared to 42.1% of those who tan more easily, and only 12% of parents of African-American children used sunscreen [38]. A 2001 Florida study found that parents of light-skinned children scored higher on UV-related knowledge than parents of children with darker skin types (74% vs. 26%, *p* < 0.05) [40]. Therefore, we recommend that some educational materials should directly target fair-skinned individuals to achieve a maximal sun-protection benefit. The identification of the Fitzpatrick skin type might be an easily applicable useful tool by caregivers and parents alike to identify higher-risk children [14,16,41].

The most common source to gather information regarding sun protection was the different means of media in our surveyed—all female participants—population at 68.4%. This is in accordance with previous findings, where it was found that the common sources of information among the surveyed individuals were television (79%), magazines (52%), newspapers (49%), health professionals (35%), and family and friends (31%). In addition, female participants showed heightened awareness of skin protection information [17]. This premise underscores the necessity and responsibility of professionals to use different media sources as means of sharing information. Today, as social media and apps are widely used, their application in primary prevention campaigns may increase campaign efficacy, although this has yet to be investigated. These channels can be used to deliver and promote the most important messages (e.g., self-skin examination method) population-wide and may be prioritized in the near future [42,43,44].

None of the participating childcare facilities had a sun safety policy, which would be much needed in order to standardize sun safety measures in centers caring for our young ones, as mentioned in former studies [19,45]. Interestingly, sun protection practices were superior in institutions with written policies [19,30,45].

Although knowledge regarding UV index was shown to be high among caregivers in both daycare centers and kindergartens, no UV index policies regulated playtime outdoors in any of the surveyed facilities. Similar findings were recently published [46]. However, caregivers were attentive in keeping children indoors during midday hours. 

Most of the institutes lack a proper shaded area, and access to good-quality sunscreen is considerably limited, as stated in a previous study [22]. We found among the surveyed kindergartens that outdoor playing areas are shaded to a lesser extent compared to the daycare centers. This phenomenon may be affected by the generally larger size of kindergartens and increased protective attitude towards younger age groups in daycare centers. While most caregivers at first thought yards belonging to child centers were properly shaded, many facilities erected sun sails and planted trees following our presentation. Shade is an important UV minimizing method and is needed in all outdoor areas where children gather (not only over “play areas” such as sandboxes). To create an effective shaded area, shade audits are promoted to assess the proper size and the location of the area for maximum protection and taking factors into account that influence shade UV protection (e.g., UV reflectivity). Such audits and documents regarding shade planning have been developed for child care centers and schools, e.g., in the United States and Australia [47,48,49]. Emphasizing the importance of this component of sun protection in educational materials and in primary prevention campaigns is important to enhance sun safety. Lack of funding to deploy shade can be a hurdle as stated in previous studies [45]. Similarly, in several locations in our study, caregivers cited that necessary changes cannot be executed due to the lack of financial support.

We also ascertained that the availability of sunscreens, sunglasses, and protective clothing was limited in the facilities. Wearing sunhats (79.7%) and applying sunscreens (48.4%) were more common in daycare centers and kindergartens than the use of sunglasses (10.5%), although the general availability of these items was significantly higher in daycare centers based on the caregivers’ judgment (*p* < 0.001). In Germany, a recently conducted study found that caregivers in nearly all institutions use sunscreen (98.8%) and sunhats (98.4%), and the usage of sunglasses was 2.4%. The results were based upon the referral of the directors of the nurseries [45]. The differences in the results between the two institutions we examined in our study might be explained by the more intense preventive attitude of caregivers (including parents) towards children under the age of three.

None of the child centers surveyed in this study supply sunscreen to children on a regular basis, due to its high cost. It falls mostly to families to provide sunscreen or, on occasion, sponsors who supply sunscreen products to facilities. According to our results, the knowledge regarding sunscreen use (re-applying and application of sufficient amount) is not appropriate among caregivers. Building sun shelters, remaining in shaded areas, and wearing hats and long-sleeved clothes are the pillars regarding sun protection, since sunscreen use alone is deemed insufficient and plants a false sense of security [22,38].

It is important to develop a general sun-safety policy that should be effectively implemented in the facilities promptly. Ideally, such a policy will not only issue guidance and set the rules regarding how and when children are taken outdoors, yet also focus on the availability of sufficient shaded areas proportionately sized to the number of children enrolled at the facility. It can be one of the first steps towards increasing sun protection awareness among governmentally funded Hungarian child care centers. Based on our results, we observed, among facilities taking care of younger children (daycare centers) the availability of sun-protection measures (e.g., hat, and sunscreen) are higher, and typically provided by the parents, the proportion of shaded areas is generally higher also compared to kindergartens.

Our study experienced several limitations, such as the reliance on self-reporting and the representativeness of the data. Even though the study was multicentric and took place in the fifth-largest city in Hungary, we cannot conclude that the sample was representative for all daycare centers and kindergartens in the region. Furthermore, the child care centers were situated in areas with average incomes, but the caregivers were not asked to divulge their place of residence. Currently, Hungarian institutions caring for pre-school-aged children encounter significant staff fluctuations, which also shows in our sample size obtained in a half-year time interval; this may also explain the stagnation and limited improvement in our results. Furthermore, privately funded institutions did not participate, although access to sun-protective measures is not only determined by financial support [45].

In conclusion, sun protection knowledge is required; however, in itself, it is not sufficient for the successful implementation of proper sun-protection behavior in kindergartens and daycare centers. According to our results, over one fourth (27%) of the caregivers did not change their sun protection habits and professed to bask in the sun despite the information presented, which is in accordance with a recent Danish study, where, after the first measures of the campaign, sunbed use was only slightly reduced (odds ratio (OR) = approx. 0.9 versus OR = approx. 0.3 after eight years of repetition) [42]. However, forty percent of the participants changed something in their habits, and the majority of caregivers passed along their information regarding sun protection to the parents. Furthermore, employees paid more attention to the sun safety of children after the presentation. These findings show a positive effect on sun-safety behavior, but most importantly, highlight the relevance of continuous education on this topic from reliable sources [39,42,50,51].

To improve and stabilize sun-protection behavior in child care centers in our region, in addition to the implementation of written institutional sun-safety policies (including shading), the availability of private and institutional funds and an incorporated repetitive educational program for caregivers is needed. In consideration of training, published literature, multi-media presentations, and online workshops with periodical mandatory testing can be used to promote and verify sufficient and lasting knowledge. The continuing education should be taught by a reliable individual, such as a well-trained nurse, repeated each spring and provided memos regarding sun protection sent to caregivers every 3–4 months. Furthermore, the motivation and involvement of parents is necessary as they play an important role in enhancing sun safety among children [7,9,22,40,50]. This study documented the limited knowledge and practices regarding sun safety, helped to increase awareness in caregivers, and allowed for recommendations for improving sun protection outcomes for children in our region.

## Figures and Tables

**Table 1:** Improvement in different knowledge traits, from baseline to follow-up questionnaire.

	Daycare Center		Kindergarten		Total		*p*-Value *
**Knowledge of sunscreen use**	Baseline (*n* = 51)	Follow-up (*n* = 56)	Baseline (*n* = 83)	Follow-up (*n* = 87)	Baseline (*n* = 134)	Follow-up (*n* = 143)	
mean performance (%)	67.2	62.5	61.5	65.1	64.0	49.2	0.222
performance 100% *n* (%)	8 (15.7)	5 (8.9)	8 (9.6)	16 (18.4)	16 (11.9)	21 (14.7)	
**Knowledge of skin type**	Baseline (*n* = 57)	Follow-up (*n* = 56)	Baseline (*n* = 96)	Follow-up (*n* = 87)	Baseline (*n* = 153)	Follow-up (*n* = 143)	
mean performance (%)	61.0	73.0	39.0	58.0	47.0	64.0	0.307
performance 100% *n* (%)	24 (42.1)	31 (55.4)	25 (26.0)	33 (37.9)	49 (32.03)	64 (44.8)	

* Mann–Whitney test.

**Table 2:** Availability of sunscreen, sunhat, and sunglasses based on the judgment of caregivers.

	Daycare Center (*n* = 57)	Kindergarten (*n* = 96)	Total (*n* = 153)
Availability of sunhat to children, *n* (%)			
Almost every child	36 (63.2)	15 (15.6)	51 (33.3)
Approx. half of the children	15 (26.3)	56 (58.3)	71 (46.4)
A few children	3 (5.3)	23 (24.0)	26 (17.0)
Availability of sunscreen to children, *n* (%)			
Almost every child	25 (43.9)	2 (2.1)	27 (17.6)
Approx. half of the children	14 (24.6)	33 (34.4)	47 (30.7)
A few children	18 (31.6)	57 (59.4)	75 (49.0)
Availability of sunglasses to children, *n* (%)			
Almost every child	0 (0.0)	2 (2.1)	2 (1.3)
Approx. half of the children	1 (1.8)	13 (13.5)	14 (9.2)
A few children	54 (94.7)	79 (82.3)	133 (86.9)

**Table 3:** Changes in sun-protection habits among caregivers in child centers.

	Daycare Center (*n* = 56)	Kindergarten (*n* = 87)	Total *n* = 143, *n* (%)
positive change in behavior	21	37	58 (40.6)
no sunbathing	16	25	41 (28.67)
stay in shade	6	10	16 (11.19)
used hat and sunglasses	7	14	21 (14.68)
used sunscreen	5	3	8 (5.59)
appropriate sunscreen use	6	11	17 (11.89)
against efforts was sunburnt	1	0	1 (0.70)
no change in behavior	35	49	84 (58.7)
never tanned before	11	19	30 (20.98)
tans anyway	16	23	39 (27.27)
no answer	0	1	1 (0.70)

## Data Availability

The data presented in this study are available on request from the corresponding author.

## Supplementary Materials

The following are available online at https://www.mdpi.com/article/10.3390/cancers13153873/s1, Figure S1: Baseline questionnaire, Figure S2: Follow-up questionnaire.
