# Supplementary material for: Results of a Primary Skin-Cancer-Prevention Campaign in Early Childhood on Sun-Related Knowledge and Attitudes in Southern Hungary
Source: Cancers (Basel). 2021 Jul 31;13(15):3873. doi: 10.3390/cancers13153873 (PMC8345575; doi:10.3390/cancers13153873)
Supplement: Supplementary file 1 [file cancers-13-03873-s001.zip › cancers-1277245-supplementary.pdf]

cancers

MDPI

Article

Results of a Primary Skin-Cancer-Prevention Campaign in Early Childhood on Sun-Related Knowledge and Attitudes in Southern Hungary

Zsuzsanna Horváth, Adriana Csernus, Péter Oláh, Rolland Gyulai and Zsuzsanna Lengyel

Baseline questionnaire

1. Have you received any training concerning sun safety behavior? If yes, within the framework of which organization?
   a. No. b. Yes, …………………………………………………..…
2. Where have you heard about the importance of sun protection? (Multiple choices are accepted.)
   a. Media (TV, radio)
   b. Newspapers, magazines
   c. Internet
   d. General practitioner
   e. Dermatologist
   f. Acquaintances
   g. Pharmacy
3. In which time frame is it not advised to stay outdoors during summer?
   a. Between 10:00-16:00 b. Between 6:00-9:00 c. Between 17:00-20:00
4. Have you ever heard about the different skin types?
   a. Yes. b. No.
5. Match the different skin types with their appropriate features?

Type I	Black skin
Type II	White skin, rarely burns and always tans well
Type VI	White skin, never tans, and always burns
Type III	Brown skin
Type V	White skin, usually burns, and tans minimally
Type IV	White skin, never burns, and always tans well

6. What defines proper sun-protection in your opinion?
   a. sunglasses, hat, sunscreen, seeking shade and wearing upper clothing
   b. hat, wearing upper clothing, seeking shade, and shoes
   c. seeking shade, sunglasses, sunscreen, and shoes
7. Does sunbathing age the skin in your opinion?
   a. Yes. b. No. c. I don't know.
8. In your opinion can solar radiation cause skin cancer?
   a. Yes. b. No. c. I don't know.
9. In your opinion what is the meaning of UV index?
   a. A number, which signals the strength of UV radiation at a particular place and time.
   b. A number marked on sunscreens.
   c. I don't know.
10. How often is it required to re-apply sunscreen?
    a. It is not necessary.
    b. Only if we go into the water
    c. Every 2-3 hours
    d. Every 6-8 hours
11. How much sunscreen should be applied on one leg?
    a. 1 teaspoon/ 6 ml b. 0.5 teaspoon/ 3 ml c. 1.5 teaspoons/ 9 ml
12. When should sunscreen be applied to the skin?
    a. On the beach, while hiking etc.
    b. 20 min. before going outside.
    c. The time of use is not important, only the fact that we use sunscreen.
13. What is the meaning of broad-spectrum sunscreen?
    a. a. Protects against UVA. b. Protects against UVA, UVB and UVC c. Protects against UVA and UVB
14. At the day- care center/kindergarten where you are employed when do you take the children outdoors in summer? (Identify the time period.)
    a.m. ……………………………………, p.m. ……………………………………

15. Above which outdoor temperature (in Celsius) do you refrain taking the children outdoors?
    ……………………………………………….…
16. Are there shady areas in the backyard of your workplace?
    a. Yes, trees give shelter against the sun.
    b. Yes, there is a roofed area in the backyard.
    c. There is no shade in the backyard.
17. Do you put hats on the children when going outdoors?
    a. Yes, always, if they have one.
    b. If they have one, and if we remember to remind them.
    c. No, it's uncharacteristic.
18. What action do you take if some children do not have hats?
    a. Parents are always reminded.
    b. Parents are reminded when we remember to do so.
    c. We don't take any actions.
19. Do you apply sunscreen lotions on children' prior to going outdoors?
    a. Yes, but only if the child has it.
    b. If and when we remember.
    c. No, there is no time for that.
20. Are parents requested to bring sunscreen into your day-care center?
    a. Yes. b. No.
21. If and when parents specifically request, do you apply sunscreen on their children?
    a. Yes, always. b. Only on children with fair skin. c. No.
22. If no, kindly explain the reason (e.g., lack of time, caregivers are few in number).
    ……………………………………………….…
23. What percentage of children have hats in your group?
    a. Almost every child. b. About the half of the children. c. Just a few children.
24. What percentage of children have sunscreen lotions in your center?
    a. Almost every child. b. About the half of the children. c. Just a few children.
25. What percentage of children have sunglasses in your group?
    a. Almost every child. b. About the half of the children. c. Just a few children.
26. Do parents provide sunscreen and sunglasses to children who are fair skinned and light haired?
    a. Yes. b. It's untypical. c. No.
27. What is your opinion regarding sun-protection, do you think it's important?
    a. Yes. b. It's exaggerated. c. No.
28. Do you want to learn more about protection against sunburn and sun screening during your education?
    a. a. Yes. b. No. c. I don't know.

Figure S1. Baseline questionnaire.

Follow-up questionnaire

1. Did you find our presentation concerning sun protection and sun safety useful?
   a. Yes, I have heard new information which to me previously was unknown.
   b. No, there was no new information.
2. Which are the characteristics regarding UV radiation? (Multiple choices are accepted.)
   a. Enhances aging and causes cancer.
   b. Strengthens the immune system.
   c. Penetrates through glass.
   d. Reflects from different surfaces, and one should consider this on the beach or when skiing.
3. Match the different skin types with their appropriate features?

Type I	Black skin
Type II	White skin, rarely burns and always tans well
Type VI	White skin, never tans, and always burns
Type III	Brown skin
Type V	White skin, usually burns, and minimally tans
Type IV	White skin, never burns, and always tans well

4. How often is it required to re-apply sunscreen?
   a. It is not necessary.
   b. Only if we go into the water
   c. Every 2-3 hours
   d. Every 6-8 hours
5. How much sunscreen should be applied on one leg?
   a. 1 teaspoon/ 6 ml b. 0.5 teaspoon/ 3 ml c. 1.5 teaspoons/ 9 ml
6. When should sunscreen be applied to the skin?
   a. On the beach, while hiking etc.
   b. 20 min. before going outside.
   c. The time of use is not important, only the fact that we use sunscreen.
7. What is the meaning of broad-spectrum sunscreen?
   a. a. Protects against UVA. b. Protects against UVA, UVB and UVC c. Protects against UVA and UVB
8. Have your sunbathing habits changed after participating in the sun safety presentation?
   a. Yes. b. No.
9. If yes, in what way? (Multiple choices are accepted.)
   a. I spend less time in the sun.
   b. I paid attention and remained in shady areas.
   c. I wear now hat and sunglasses.
   d. I didn't use sunscreen previously, however this summer I consciously applied it prior to going outdoors.
   e. Earlier I used sunscreen with inappropriate SPF, however this summer I knew which to pick.
   f. I tried to increase my level of attention, however, I got sunburned several times.
   g. Other: ………………………………………………..….
10. If not:
    a. I have never stayed in the sun.
    b. I did not change my habits, since I like to sunbath, and I did not get sunburnt, especially when using sunscreen.
    c. I did not think it was necessary to change my habits since sun safety should not be taken seriously.
11. I shared new information regarding sun safety with (Multiple choices are accepted.):
    a. My family.
    b. My friends.
    c. We shared the useful information with other colleagues who could not attend the presentation.
    d. No.

12. We paid more attention to sun safety regarding children enrolled in our day-care center/ kindergarten.
    a. Yes. b. No.
13. If yes, what changed? (Multiple choices are accepted.)
    a. We paid more attention and kept children in shady areas while outdoors.
    b. We distributed sunhats to whomever owned one.
    c. We applied sunscreen on those who brought it from home.
    d. We applied sunscreen onto everyone.
14. Were there any favorable changes in the day care center/ kindergarten regarding sun safety?
    a. Yes, we planted trees in hopes of establishing larger areas of shade.
    b. Yes, we erected shade sails in the yard/ above the sand boxes/ and pools.
    c. We could not afford it; however, it is considered a necessity.
    d. No, everything is appropriate in our institute.
15. Did you draw the attention of parents to the major points regarding sun safety you heard during the sun safety presentation?
    a. Yes, we draw parental attention to the dangers and to the possible methods regarding sun protection (long sleeved, loose clothing, broad brimmed sunhat, sunglasses with UV filter, staying in shade, sunscreens…. etc.).
    b. We only informed the parents whose children are fair skinned for the time being.
    c. There was no time to so.
    d. We believe, it is highly important, so we plan changes in the future/ we will pay attention to do so in the near future.

Figure S2. Follow-up questionnaire.
